# 1D Measurement of Sodium Ion Flow in Hydrogel After a Bath Concentration Jump

**DOI:** 10.1007/s10439-015-1293-8

**Published:** 2015-03-19

**Authors:** R. W. Roos, L. Pel, H. P. Huinink, J. M. Huyghe

**Affiliations:** 1 Department of Biomedical Engineering, Eindhoven University of Technology, Eindhoven, The Netherlands; 2 Department of Applied Physics, Eindhoven University of Technology, Eindhoven, The Netherlands; 3 Department of Mechanical and Biomedical Engineering, Eindhoven University of Technology, Eindhoven, The Netherlands

**Keywords:** Na-NMR, Polyacrylamide, Deformation–diffusion coupling, Quadriphasic model

## Abstract

NMR is used to measure sodium flow driven by a 1D concentration gradient inside poly-acrylamid (pAA) hydrogel. A sodium concentration jump from 0.5 M NaCl to 0 M NaCl is applied at the bottom of a cylindrical pAA sample. The sodium level and hydrogen level are measured as a function of time and position inside the sample for 5 days. Then a reversed step is applied, and ion flow is measured for another 5 days. During the measurement, the cylindrical sample is radially confined and allowed to swell in the axial direction. At the same time, sodium and moisture in the sample are measured on a 1D spatial grid in the axial direction. A quadriphasic mixture model (Huyghe and Janssen in Int J Eng Sci 35:793, 1997) is used to simulate the results and estimate the diffusion coefficient of sodium and chloride. The best fit results were obtained for D$_{Na^+} = 1.15\times 10^{-5}$ cm^2^/s and D$_{Cl^-} = 2.15\times 10^{-5}$ cm^2^/s, at 25 degrees centigrade. Different time constants were observed for swelling and deswelling.

## Introduction

Biological tissues and cells are in continuous renewal and therefore need constant supply of nutrients and removal of waste products. Transport is caused by gradients in pressure, ionic concentration, electrical potential or temperature. In practice, often a combination of them is involved. In addition, these gradients are often affected by the transport parameters and the swelling state of the material. Hence, frictional phenomena in biology are very intricate. Many authors use hydrogel as a model material to study coupled diffusion.^7^–^10^,^14^,^17^, ^19^

Among all extracellular ions, sodium has the largest concentration. Because of its high affinity to water, it tends not to bind to the negatively charged macromolecules, but rather build double layers around it. Therefore, sodium plays a vital role in the water binding capacity of the extracellular space. In the intervertebral disc, sodium concentration is a measure of the fixed charge density ($c^{fc}$) of the tissue which in turn is a measure of disc degeneration.^1^,^2^,^5^,^13^,^18^ The ocular lens relies on internally directed ion and water flow for its circulation.^22^ Hence, a thorough understanding of sodium diffusion in presence of negatively charged macromolecules is essential in the context of the physiology of the disc, the eye as well as in tissue-engineering applications.

Because the water binding capacity of sodium is the driving force in the swelling mechanism in extracellular space, diffusion of sodium can not be decoupled from swelling and deformation. Hence thee is a pressing need for reliable modelling of the deformation-dependent diffusion of ions inside gels and biological tissues. Some of the models describe the phenomenon on the pore level,^3^ others tackle the issue on the tissue/gel level.^11^,^21^,^23^ Huyghe and Janssen have developed a quadriphasic model describing the coupling between diffusion and swelling in a gel-like medium.^11^ The aim of this study is to measure sodium diffusion in hydrogel during swelling and to verify the validity of the quadriphasic model. Given the experience with sodium NMR in various labs around the world,^15^,^24^ NMR has been chosen as a carrier for this experimental study.

## Method and Materials

### Sample Preparation

The material consists of a hydrophilic copolymer gel, which has been synthesized by means of polymerization of acrylic acid (AA) and acrylamide (AAm) monomers. After polymerization, the sample is cut at 9 mm length, blotted, weighed and placed in a 0.5 M NaCl bath. The first week after preparation, the concentration bath is refreshed three times to wash the gel from initiator and unreacted monomers. After three weeks, the sample is used for the sodium diffusion experiment.

Between preparation and the beginning of the experiment, the sample volume has increased by a factor $J_1=5.6$. This swelling is accompanied by an increase of the fluid volume fraction $N^f$ and a decrease of fixed charge density $c^{fc}$ per unit fluid volume:

1 $$\begin{aligned} c^{fc} = \frac{c^{fc}_0}{N^f} \end{aligned}$$

In the above equation, $N^f$ is the current fluid volume divided by the initial mixture volume. The initial fixed charge density $c^{fc}_0$ is taken 0.8 (mol/l), this yields $c^{fc}$ = 0.14 (mol/l). In addition, the fluid fraction $n^f_1=J_1N^f_1$ at the start of the experiment is calculated from the fluid fraction $N^f_0$ and the sample volume $V_0$ just after preparation, the absorbed water between preparation and experiment $V^f_{abs}$, and the final sample volume $V_1$:

2 $$\begin{aligned} n^f_1=J_1N^f_1 = \frac{ N^f_0\times V_0 + V^f_{abs}}{V_1}, \end{aligned}$$

where the subscript 1 refers to the start of the experiment, the subscript 0 to the preparation, $n^f$ refers to fluid volume per unit current mixture volume and $N^f$ refers to fluid volume per unit initial mixture volume. If we use $N^f_0$ = 0.8, the measured volume yields $n^f_1$ = 0.97 at the start of the experiment, and increases less than 10% during the experiment.

### Technical NMR Details

**Figure 1:**
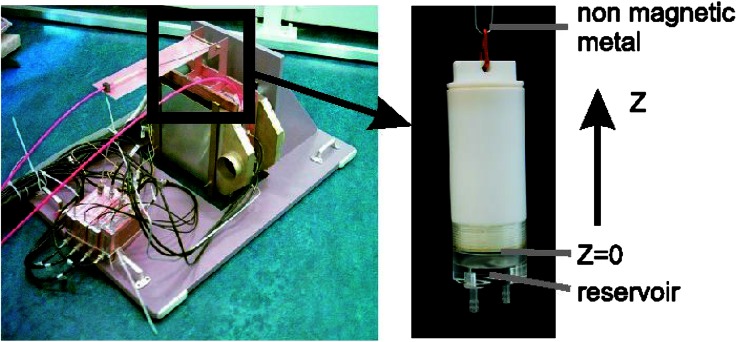
A picture of the inset for quasi-simultaneous measurement of $^{23}$Na and $^{1}$H (left), and one of the cylindrical teflon sampleholder (right). At the bottom of the sampleholder ($z=0$), a glass filter separates the pAA sample from the small chamber through which a salt solution is flushed.

The sampleholder consists of a Teflon chamber and a Perspex reservoir. It is separated by a porous glass plate and connected on top by a non-magnetic metal wire. This reservoir of 5 mm height and 25 mm diameter has two holes in the bottom with connection ends for a tube. Before the cylindrical pAA sample is placed in the sampleholder, it is blotted and weighed to determine the swelling degree at $t=0$, when the sample is in equilibrium with a 0.5 M NaCl external bath concentration. During the experiment, the cross-sections of the sample are subsequently probed by a $^{1}$H-sensor and a $^{23}$Na-sensor on a certain spatial grid in the axial direction. With the current parameter settings (Table 1), the measurement of an entire profile takes about 3 h (Fig. 1).

**Table 1:** Parameter values in the Na-NMR diffusion measurements.

	Hydrogen	Sodium
Repetition time (s)	5	0.25
Echo time ($\upmu$s)	500	500
Window width ($\upmu$s)	400	400
$z$-gradient (mT/m)	80	70
Number of averages	6	600
Measurement time per profile (h)	0.5	2.5

### One Dimensional Response of a Bath Concentration Jump

**Figure 2:**
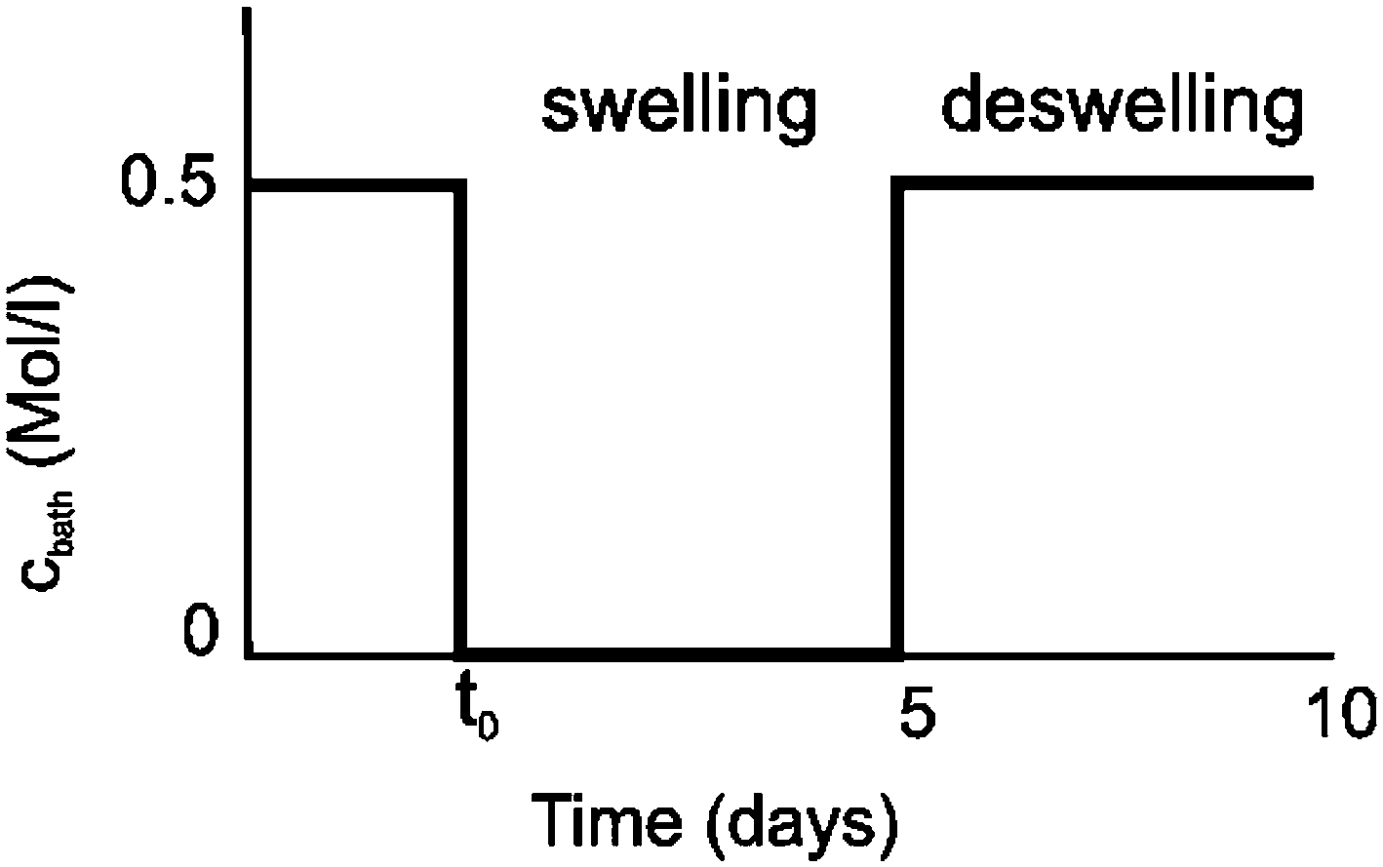
The time course of bath concentration in the transient experiment. The experiment starts at t$_0$. The first—*swelling*—period, the pAA sample is in contact with demineralized water and the second—*deswelling*—period with 0.5 M NaCl.

The measurement of sodium transport in pAA is done by applying a concentration gradient and measuring the consequent ion levels inside the sample. The easiest way to obtain a concentration gradient under well controlled boundary conditions, is by applying a jump in bath concentration. This is implemented by flushing solution from a 2 l container through the sampleholder reservoir. The reservoir is in contact with the sample only along its base. First the bath concentration is brought to 0 M, and after five days the concentration is brought back to 0.5 M NaCl (Fig. 2). In addition, the pAA sample is confined in a teflon tube (’sample cylinder’) with 16 mm radius, which is placed in the teflon chamber of the sampleholder. Grease is used to prevent leakage between the sample cylinder and the sampleholder.

## Results

### Sodium Diffusion Caused by a Concentration Step

**Figure 3:**
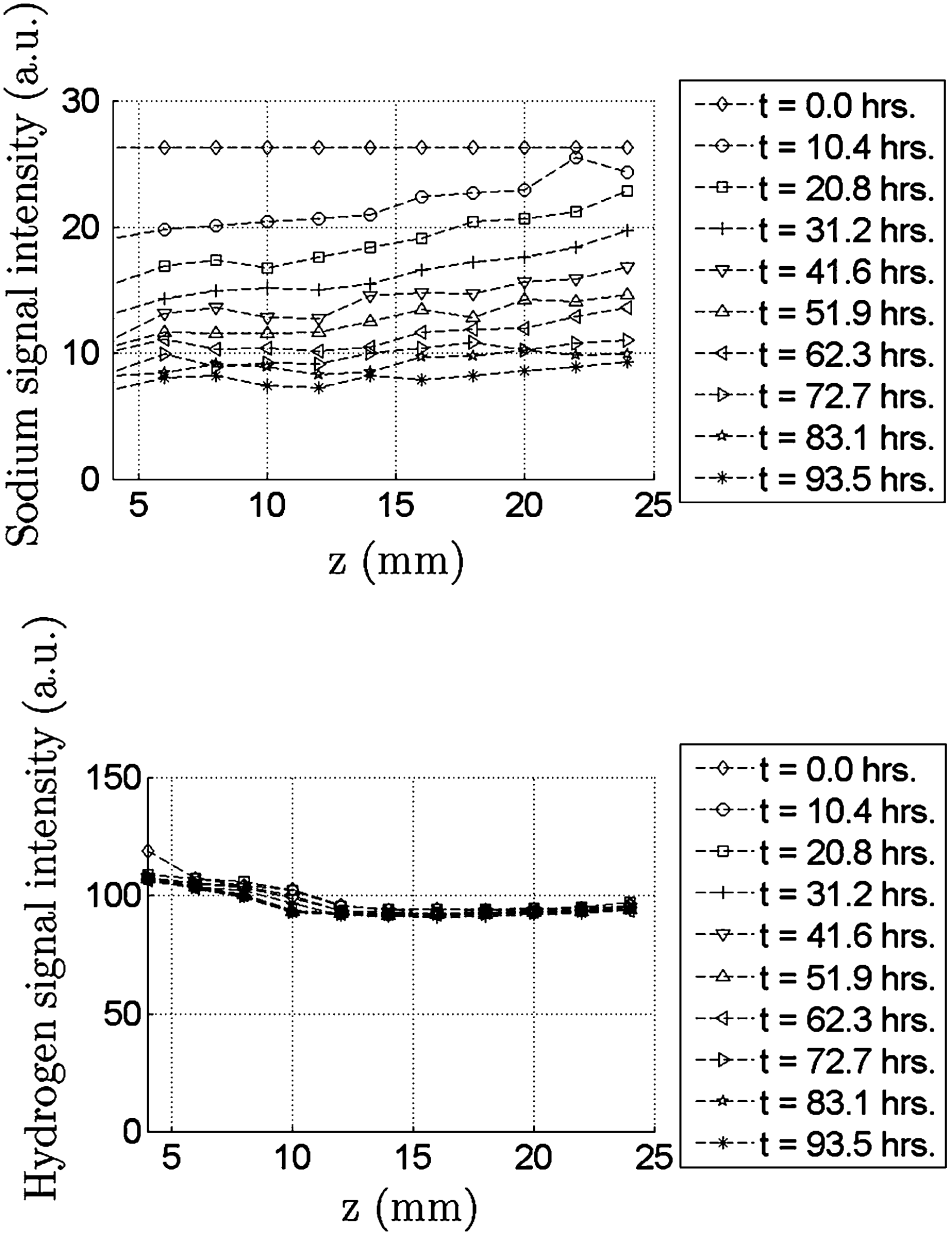
The course of sodium profiles (top) and hydrogen profiles (bottom) during the swelling period (Fig. 2). The profiles are constructed from an interpolation of the raw data at an equidistant time grid. Exchange of water and sodium between the pAA sample and the external reservoir only occurs *via* the bottom of the sample at $z=0$. The variation of the hydrogen signal during the swelling period is less than 5%.

**Figure 4:**
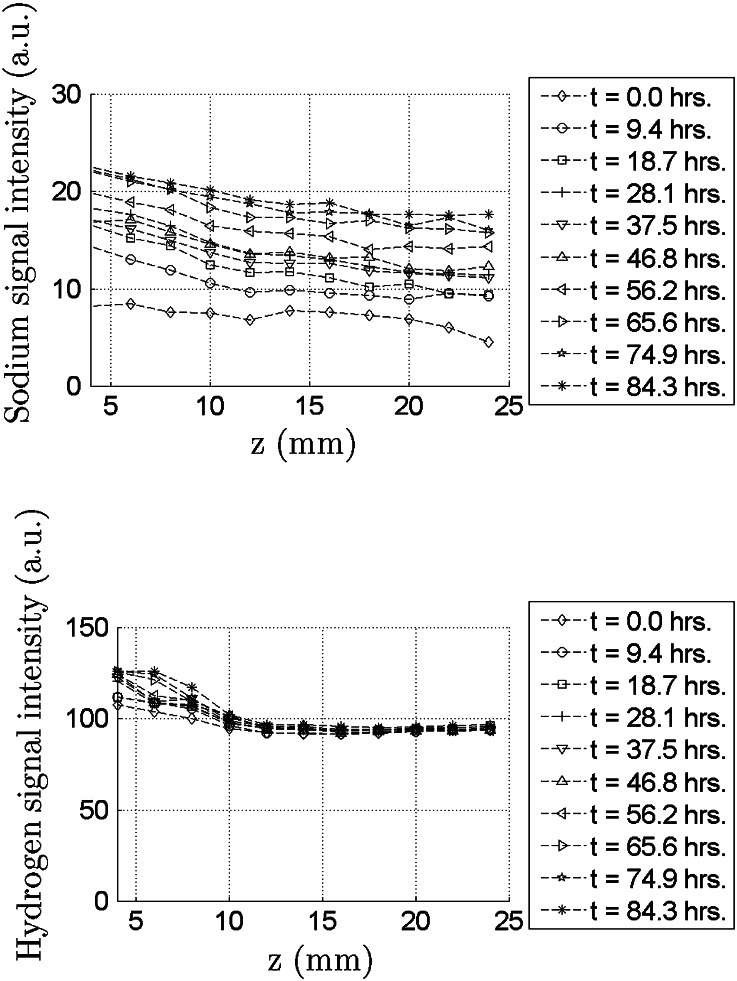
The course of sodium signal during the deswelling period (Fig. 2). Exchange of water and sodium between the pAA sample and the external reservoir only occurs *via* the bottom of the sample at $z=0$. The variation of the hydrogen signal during the swelling period is smaller than 5%.

1D transport of sodium through radially confined pAA samples is caused by a bath concentration jump at one end of the sample. This process is accompanied by sample swelling. Subsequent measurements series of $^{23}Na$ and $^1H$ are done on a spatial grid in the axial direction. Each measurement series takes 3 hours, which is a long period compared to the sodium diffusion rate in pAA. Therefore, time profiles are calculated by interpolation of raw data on an equidistant time grid to give an unambiguous representation of the measured sodium diffusion. The results for the swelling period are displayed in Fig. 3 and for the deswelling period in Fig. 4.

At the bottom of the sample ($z<5 {\rm mm}$), a peak in hydrogen is an artefact caused by water between the perspex reservoir and the teflon sampleholder. Deformation of the sample during the experiment is caused by in- and outflow of water, and alters the fluid fraction of the gel. This variation of $^1$H signal starts at the bottom of the sample, but the peak artefact defeats good recovery of this variation (Figs.  3, 4). Because the hydrogen level remains the same in other parts of the gel, we take the sodium signal as a measure of sodium concentration.

#### Material Parameters

The measurements have been simulated with a quadriphasic mixture model,^11^ which was implemented in Matlab for 1D simulations by Frijns.^6^ Former experiments yielded the following material parameters for pAA:

- the aggregate modulus $H$: 300 kPa^20^
- the hydraulic permeability $K$: ${1.2\times 10^{-16}\ {\rm m}^4 {\rm N}^{-1}s^{-1}}$ 12,20

Lanir *et al*.^14^ have performed diffusion measurements on pAA at different swelling situations (i.e.: different fluid fractions). Their proposed dependence of the sodium and chloride diffusion coefficients on fluid fraction $N^f$ at 4 $^{\circ }$C has been used to fit a combination of diffusion coefficients:

3 $$\begin{aligned} D_{Na^+}&= (1.24\times N^f - 0.57)\times 10^{-9} \ \ {{\rm m}^2/{\rm s}}\end{aligned}$$

4 $$\begin{aligned} D_{Cl^-}&= (2.49\times N^f - 1.34)\times 10^{-9} \ {{\rm m}^2/{\rm s}} \end{aligned}$$

#### Relative Concentration Changes

Given experimental data from earlier work,^20^ we assume that the hydraulic permeability and the aggregate modulus does not depend on sodium concentrations, as the dependence of diffusion coefficients on water content are already a major numerical challenge. As a matter of fact, for the small aggregate modulus of pAA and the large applied bath concentration jump, the simulation did not converge. Therefore, we introduce the relative concentration change, which transforms the actual simulated or measured concentration to a value between 0 and 100%:

5 $$\begin{aligned}  {\rm Swelling:}\qquad \hat{c}^+_{rel}&= \frac{\hat{c}^+(t) - \hat{c}_f}{\hat{c}^+_1 - \hat{c}^+_f},\end{aligned}$$

6 $$\begin{aligned}  {\rm Deswelling:}\qquad \hat{c}^+_{rel}&= \frac{\hat{c}^+(t) - \hat{c}_1}{\hat{c}^+_f - \hat{c}^+_1}, \end{aligned}$$

where ĉ(t) is the concentration as a function of time multiplied with the local water content $JN^f$ (simulation), or the measured sodium signal (measurement) and the subscripts $f$ and $1$ denote the final bath concentration and the concentration at $t=0$ respectively. The relative concentration change allows us to compare time courses of sodium signal after bath concentration jumps of different strengths. We assume linear dependence of electrochemical potential on sodium concentration^11^ so that the initial and end points of the non-linear curve of the relationship are linearly interpolated. Therefore, the time course of the relative concentration change is independent of the bath concentration jump under no-swell conditions. If we do allow swelling and deswelling, the time course of the relative concentration change becomes different for different absolute values of the concentrations. However, in our measurement, the sample (de)swelling remained within 3 mm which is about 10%.

In order to soften non-linearities in the simulation, we applied a bath concentration jump from 0.5 M to 0.3 M for 5 days, and vice versa. In order to calculate relative concentration changes, the equilibrium concentration values have to be estimated. The equilibrium values for the simulation are calculated by the conventional expression of ion partitioning based on Donnan osmosis and electroneutrality:

7 $$\begin{aligned} c^+_g = \frac{1}{2}\left( -c^{fc}+\sqrt{(c^{fc})^2 + 4c_b^2}\right) , \end{aligned}$$

where the subscript *g* denotes *gel* and *b* refers to *bath*.

For estimation of corresponding measurement values, we separate the *’swelling phase’* and the *’deswelling phase’* (Fig. 2). We assume that the sample is in equilibrium with 0.5 M NaCl before the first part of the measurement starts. The first measurement of the first measured position is hardly affected by ion diffusion, and therefore the initial sodium value in this point is taken as a 100% reference. For determination of the 0% reference value, the sodium signal during the deswelling phase has been depicted against $z_d/\sqrt{t}$ and an errorfunction is fitted on this data. A mathematical description of the tracer diffusion in one-dimensional, semi-infinite media is given in:^4^

8 $$\begin{aligned} S(t) = S_0  {\rm erf}\left( \frac{z_d}{2\sqrt{Dt}}\right) , \end{aligned}$$

with S(t) is the NMR sodium signal and $z_d$ is the diffusion depth. The asymptote at $z_d/\sqrt{t}\rightarrow \infty$ corresponds to the equilibrium sodium concentration with 0 M bath concentration and is used as 0% reference.

#### Diffusion Coefficients

The relation between D$_{Na^+}$ and D$_{Cl^-}$ is deduced from 4:

9 $$\begin{aligned} D_{Na^+} = (0.498\times D_{Cl^-} + 0.0774)\times 10^{-5} \  {\rm cm^2/s} \end{aligned}$$

Diffusion coefficients for the simulation of the measurement were varied gradually until a reasonable fit was found. In order to do so, a measure for the total error is introduced. This error measure is defined as the sum of the absolute differences between measurement and experiment at all places in the sample and at all times of the measurement. Therefore, the calculated sodium concentration by our model has been interpolated to the same time grid and position grid of the measurement.

**Figure 5:**
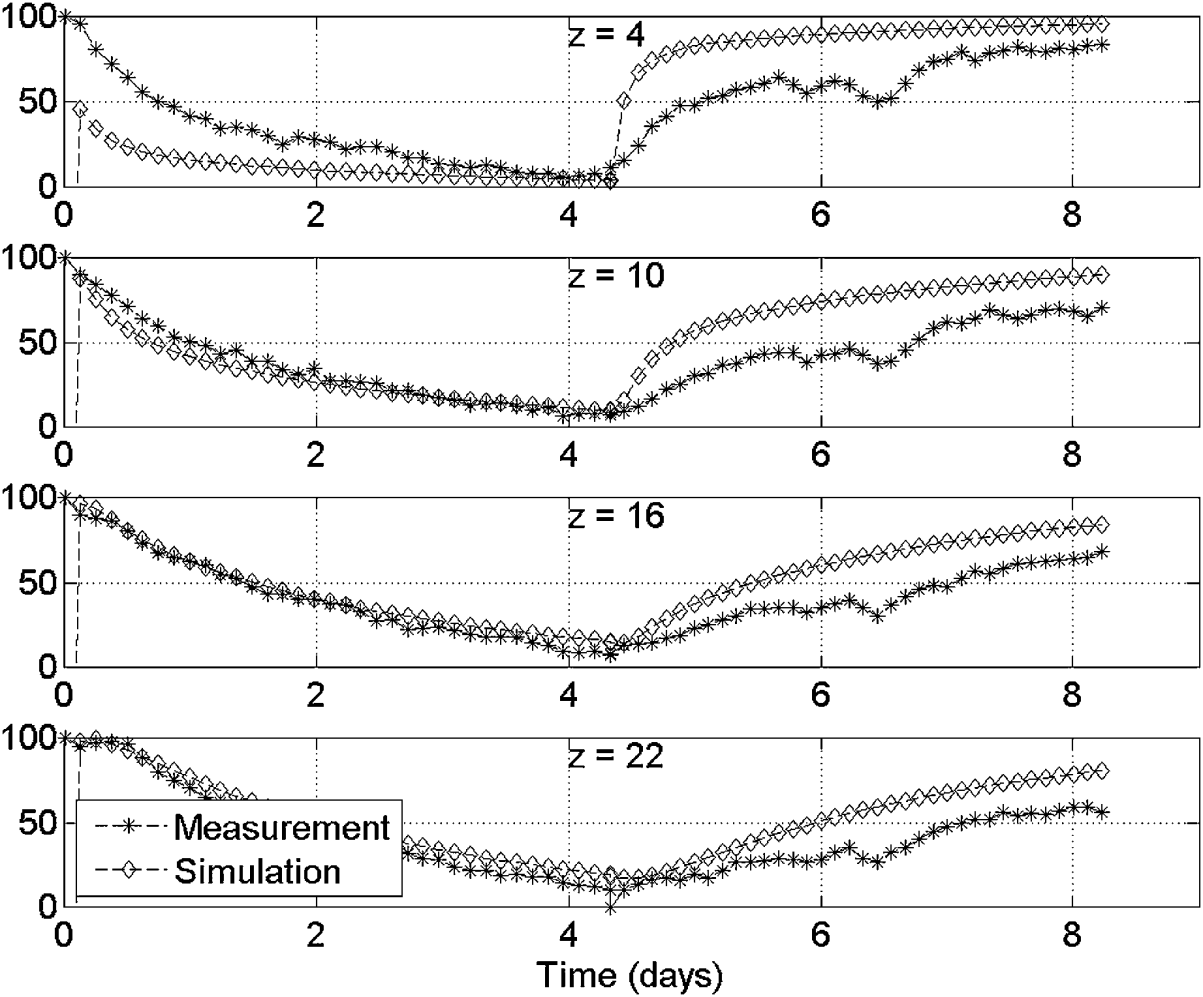
Time course of the relative sodium level at several planes perpendicular to the axis. The relative sodium level is scaled between the equilibrium level at the low concentration and the high concentration. For the model D$_{Na^+}$ = $1.15\times 10^{-5}$ cm$^2$/s and D$_{Cl^-} = 2.15\times 10^{-5}$ cm$^2$/s is used.

**Figure 6:**
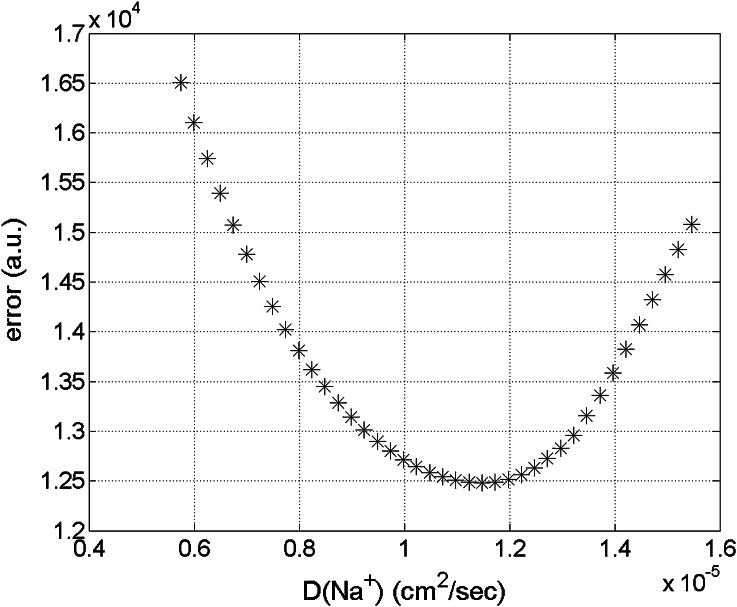
The total difference between experiment and simulation is minimized for D$_{Na^+} = 1.15 \times 10^{-5}$ cm$^2$/s and D$_{Cl^+} = 2.15 \times 10^{-5}$ cm$^2$/s.

Minimizing the error, yields a converging minimum for the diffusion coefficients of sodium and chloride (Fig. 6):

- D$$_{Na^+}= 1.15\times 10^{-5}  {{\rm cm}^2/{\rm s}}$$
- D$$_{Cl^-}= 2.15\times 10^{-5}  {{\rm cm}^2/{\rm s}}$$

## Discussion

One dimensional sodium diffusion in pAA is measured with NMR. The results are used to fit the diffusion coefficient of sodium and chloride with a quadriphasic mixture model. We obtain a good correspondence between measurement and simulation. The values of the fitted diffusion coefficients are D$_{Na^+}= 1.15\times 10^{-5}  {{\rm cm}^2/{\rm s}}$ and D$_{Cl^-}2.15\times 10^{-5}  {{\rm cm}^2/{\rm s}}$ Compared to the predicted value of^14^ (D$_{Na^+} = 0.63\times 10^{-5}  {{\rm cm}^2/{\rm s}}$ and D$_{Cl^-}1.08\times 10^{-5}  {{\rm cm}^2/{\rm s}}$), the fitted coefficients are a factor 2 higher. This factor can be explained by temperature dependence of diffusion coefficients (which is about 2–3% per degree centigrade above 25°C^25^). Because the NMR measurements induce internal sample heating, the—uncontrolled—temperature during the NMR measurement was some degrees above room temperature. Extrapolation of Lanir’s results to the temperature range of the NMR measurement yields similar values for diffusion coefficients. Also the dependence of the fit on the aggregate modulus—H—and the hydraulic permeability—K—were explored, but these factors yield relatively small contributions to the fitting error.

Figure 5 shows a comparison between the simulation and the experiment in several cross-sections as a function of time. Except for the borders of the sample, the simulation results are in good correspondence with the measured data. At the borders, the effect of limited spatial resolution of the sodium probe disturbs the signal. We do not expect the grease to affect the results adversely, as the grease does not contain any sodium and is hydrophobic. Furthermore, we observe a consistent underestimation of the concentration for the downward concentration jump, and the consistent overestimation of the concentration for the upward concentration jump. Earlier results from our group indicates similar asymmetries^20^ for the displacement tracings. In the first case (swelling) the diffusion coefficient is underestimated, and in the latter case (deswelling) it is overestimated. This asymmetry may be caused by an interplay between sodium concentration, electric double layer size, difference between hydraulic permeability and ion self-diffusion, and the fluid fraction in hydrogel. During swelling, water flows in the positive $z$-direction, while sodium flows in the opposite way. Therefore, the sodium concentration rapidly decreases close to the border between bath and gel, while at the same time the water content there increases. Given the increasing diffusion coefficient with increasing water content, the diffusion coefficient increases close to the border. If we consider deswelling, the situation is reversed. The water content close to the sample boundary is decreased, yielding a lower local diffusion coefficient and blocking sodium and chloride transport in the sample. The dependence of the diffusivities on water content are introduced in the quadriphasic model. So we expect the model to be able to reproduce this asymmetry, provided that the full extent of the dependence of diffusivities is accounted for. Because the concentration jump has not been fully enforced in the modelling, only part of the non-linearity induced by Eq. (4) has been accounted for. This is a possible explanation for the discrepancy between model and experiment. The results indicate that sodium-NMR is a suitable probe for measurement of sodium transport. Sodium-NMR yields a transparent view of sodium diffusion in a swelling medium. The quadriphasic model overestimates the diffusion coefficient in the downward concentration jump, whereas it underestimates the diffusion coefficient in the upward concentration jump (Fig. 5). This suggests that the frictional behavior of hydrogel equilibration strongly depends on the local properties of the gel. In particular, the water content strongly affects diffusional properties. In order to handle the strong non-linearities of the quadriphasic model in large deformations, it is advisable to replace the present formulation by a Raviart–Thomas formulation that respects local mass balance at all times.^16^
